# Therapeutic efficacy of programmed spatial anatomy of the myopectineal orifice in total extraperitoneal hernioplasty: a retrospective study

**DOI:** 10.1038/s41598-023-29671-0

**Published:** 2023-02-15

**Authors:** Lin Zhuang, Yuanjiu Li, Wei He, Xiaodong Zhou, Yan Chen, Xiaozhong Wang, Bo Wang, Xuezhong Xu, Kejia Wu, Qiutao Zhang, Dong Xi, Yunjie Lu

**Affiliations:** 1 Department of General Surgery, Wujin Affiliated Hospital of Jiangsu University and The Wujin Clinical College of Xuzhou Medical University, Changzhou, Jiangsu China; 2 The First People’s Hospital of Changzhou, The Third Affiliated Hospital of Soochow University, Changzhou, Jiangsu China; 3 Department of General Surgery, Wujin Fourth People’’s Hospital, Changzhou, Jiangzhou China; 4 Africa Hepatopancreatobiliary Cancer Consortium (AHPBCC), Mayo Clinic, Jacksonville, FL US

**Keywords:** Outcomes research, Medical research, Clinical trials

## Abstract

This study aimed to investigate the therapeutic efficacy of programmed spatial anatomy of myopectineal orifice technique in laparoscopic total extraperitoneal hernioplasty (TEP) surgery. A total of 121 adult male patients with unilateral inguinal hernias who underwent TEP in the Department of General Surgery, Wujin Hospital, affiliated with Jiangsu University, from January 2019 to December 2020 were selected. Patients were divided into the procedural (63 cases) and traditional groups (58 cases) according to the surgical methods adopted. The procedural group underwent programmed spatial anatomy of the myopectineal orifice combined with TEP, and the traditional group underwent traditional TEP. The perioperative evaluation indicators and postoperative complications were observed and compared between the two groups. Compared with the traditional group, the time of handling hernia, the intraoperative operation time, intraoperative blood loss, postoperative ambulation time, and postoperative hospital stay in the procedural group were significantly reduced (*P* < 0.05). The incidence of postoperative complications such as sensory nerve abnormalities and chronic pain was significantly decreased (*P* < 0.05), and the total incidence of complications in the procedural group was significantly lower than that in the traditional group (*P* < 0.05). While there was no significant difference in postoperative incision infection (*P* > 0.05). The programmed spatial anatomy of the myopectineal orifice can significantly improve the treatment outcome of TEP, significantly improve the patients' intraoperative and postoperative indicators, and reduce the incidence of postoperative complications. It is worthy of being promoted among young physicians and basic hospitals.

## Introduction

Inguinal hernia is a common disease caused by the relaxation of the transverse abdominal fascia and weakness of the myopectineal orifice^1^. With the promotion of precision medicine and the continuous improvement of medical device technology, laparoscopic surgery has gradually replaced traditional surgical methods for treating inguinal hernia^2^. Laparoscopic total extraperitoneal hernioplasty (TEP), a mainstream laparoscopic procedure, pays special attention to the anatomy of the myopectineal orifice. A successful TEP can improve the size of the space and outline the myopectineal orifice, which is beneficial in completely covering the entire myopectineal orifice area with the mesh. At present, the traditional TEP method follows the guiding philosophy of open surgery; only the surgical approach and visual field are changed, not the surgical space and hierarchical structure^3,4^. The limitations of traditional TEP surgery include the ambiguity of establishing the extraperitoneal space, the disorder of operative procedures, incompleteness of the anatomy of the myopectineal orifice, which could increase the injury probability of important vessels and tissues in operation, and the incidence of intra- and postoperative complications.

Programmed spatial anatomy of myopectineal orifice TEP surgery is a programmed decomposition of TEP surgery according to the anatomical characteristics of the myopectineal orifice, which can systematically and hierarchically divide the extraperitoneal space into four spatial regions, and confirm the key points and difficulties of operation in each spatial area, to ensure that the operation is clear and orderly. At present, the concept of programmed spatial anatomy of myopectineal orifice has been widely recognized^5^, which is consistent with the concept of modern precision medicine. We adopted this novel surgical approach to treat hernias to ease the operative procedure in TEP operations. This modification effectively shortens the operation time, postoperative ambulation time, and postoperative hospital stay and reduces surgical trauma and the incidence of complications.

We conducted a retrospective cohort study to compare the application effect of programmed spatial anatomy of myopectineal orifice technique in laparoscopic TEP surgery with TEP surgery alone in repairing inguinal hernias to promote the popularization and application of programmed spatial anatomy of myopectineal orifice TEP surgery.

## Materials and methods

### Patients

A total of 197 adult males with unilateral inguinal hernias who underwent TEP surgery in Wujin Hospital, affiliated with Jiangsu University from January 2019 to December 2020 were initially enrolled. Based on the inclusion and exclusion criteria, 76 patients were excluded and clinical data of 121 adult male patients were collected. The patients were divided into the procedural (63 cases) and traditional groups (58 cases) according to different surgical methods.

The inclusion criteria were as follows: (1) adult male patients above 18 years old; (2) unilateral reducible indirect inguinal hernia had been confirmed clinically; (3) the operation was completed by our team from January 2018 to December 2020. The exclusion criteria were: (1) a history of lower abdominal surgery; (2) unwilling to participate (e.g., refusing to cooperate with treatment); (3) direct hernia with or without indirect hernia.

This study was reviewed and approved by the Ethics committee of The Wujin Affiliated Hospital of Jiangsu University. All patients participating in this study signed an informed consent form and all methods were performed in accordance with the relevant guidelines and regulations.

### Surgery methods

All patients underwent general anesthesia with endotracheal intubation and were placed in a supine position with their heads and feet held high.

**Figure 1:**
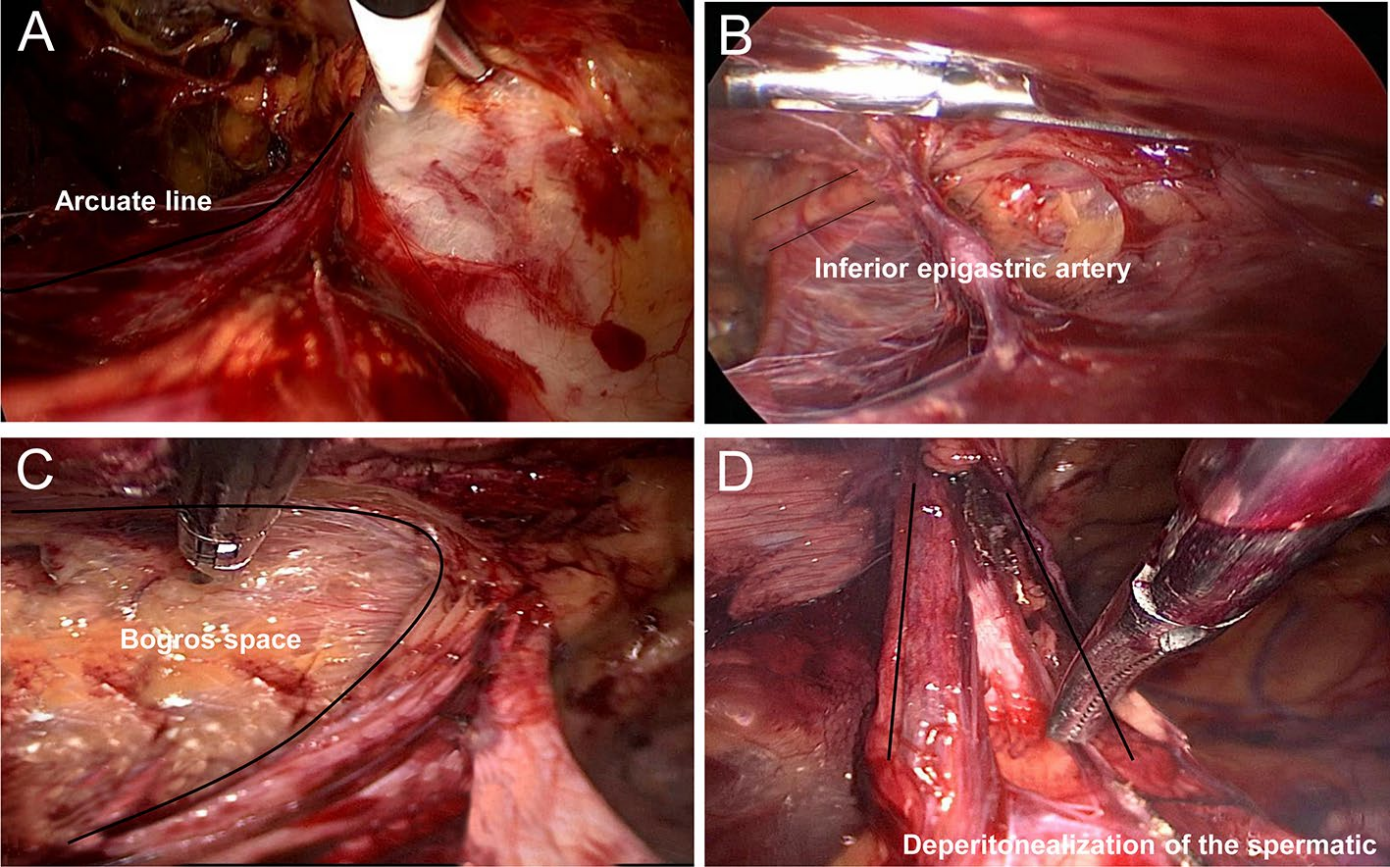
Important anatomical landmarks within each space of the "Programmed Spatial Anatomy of the Myopectineal orifice Region." (**A**) The lower edge of the arcuate line; (**B**) the inferior epigastric artery; (**C**) the Bogros space; (**D**) the spermatic cord after deperitonealization.

**Figure 2:**
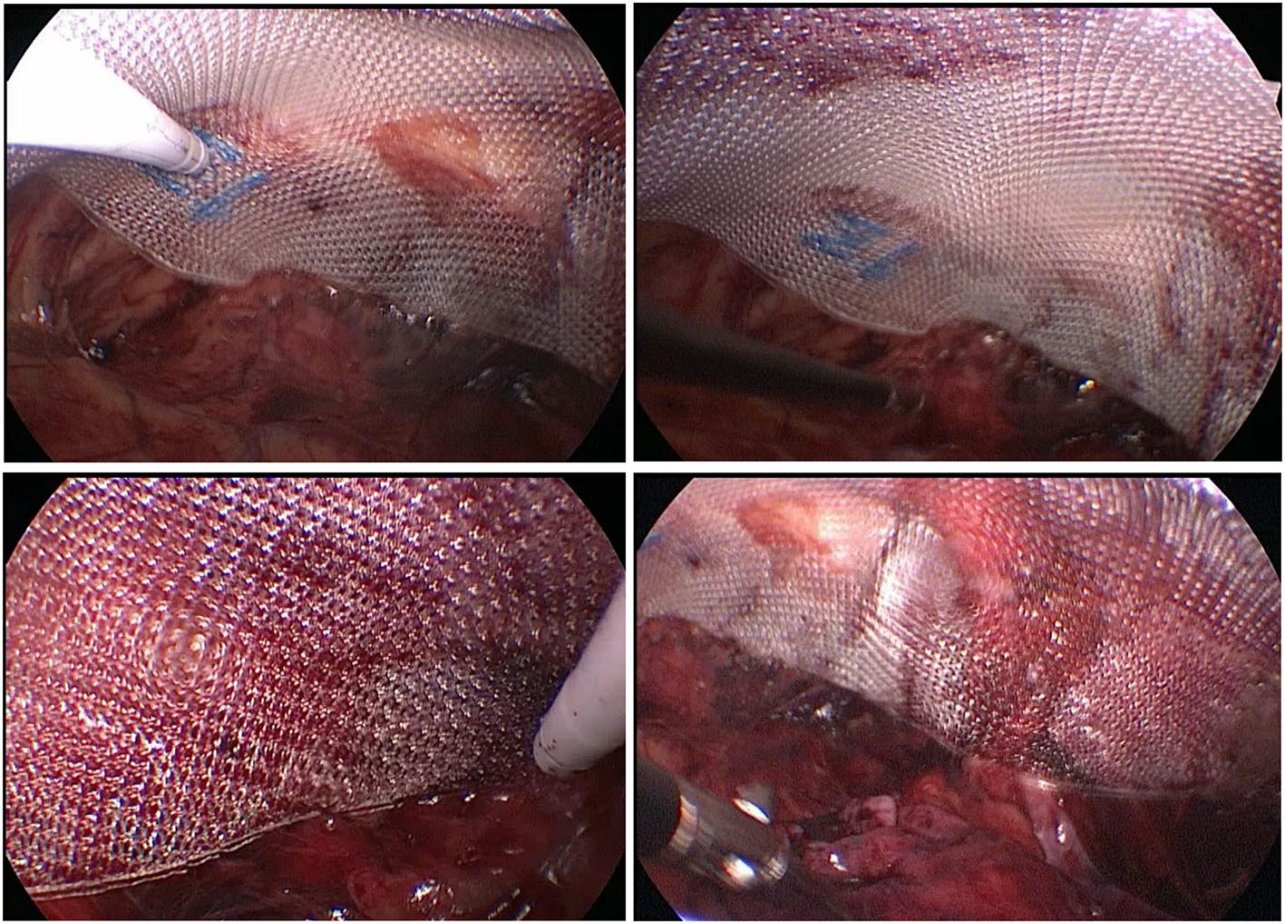
Intraoperative 3D mesh fixation.

The procedural group used the "programmed spatial anatomy of myopectineal orifice" technique to construct the extraperitoneal space as follows: an incision of about 1.0 cm was made on the lower edge of the umbilicus slightly closer to the affected side. The skin and subcutaneous tissue were incised to expose the posterior rectus abdominis sheath. The septum of the linea alba, the posterior sheath of rectus abdominis, and the preperitoneal space on the affected side were bluntly separated by oval forceps to synthesize the "first space" of the myopectineal orifice. The main anatomical structure of this space is the arcuate line (Fig. 1A). A 5-mm trocar was directly punctured into the preperitoneal space at about the upper 1/3 and lower 1/3 of the line between the umbilicus and the pubis. Carbon dioxide gas was injected, and the pressure was maintained at about 10 mmHg. The preperitoneal space on the affected side was separated with an electric hook or ultrasonic knife, the transverse abdominal fascia was fully opened, and the pubic comb ligament and pubic tubercle were exposed. This was the "second space" of the myopectineal orifice, mainly covering the direct hernia triangle, the direct hernia ring, and part of the upper medial area. The inferior epigastric artery was found after bluntly separating outward along the "second space" (Fig. 1B). The Bogros space was opened outward (Fig. 1C), and the iliac fossa space was separated to expose the inner ring of the indirect inguinal hernia. This was the "third space" of the myopectineal orifice area, which mainly covered the inner ring of the indirect inguinal hernia, the triangle of indirect hernia, the triangle of pain, and part of the lateral area. For patients with an indirect inguinal hernia, the hernia sac was completely pulled out to fully separate the hernial sac from the spermatic vessels, vas deferens, or uterine round ligament (Fig. 1D). This was the "fourth space" of the myopectineal orifice, which mainly covered the dangerous triangle and anatomical structure of the lower half of the myopectineal orifice. The spermatic cord was deperitonealized, and each spatial area was fully fused to form a complete spatial structure of the myopectineal orifice. The Bard 3DMax polypropylene mesh (Shanghai Hanrong Medical Instrument Co., LTD, China) was placed in the preperitoneal space, fully covering the myopectineal orifice area and fixed with or without medical glue (Fig. 2). After fully releasing the gas, the trocar was pulled out, and the surgical incision was sutured^6^.

For patients in the traditional group, holes were respectively punctured at the three equal points of the umbilicopubic line under the guidance of laparoscopy, and a 5 mm trocar was implanted in each hole. The extraperitoneal space was constructed from medial beyond the midline 2 cm, lateral to the iliac crest, below beyond the pubic symphysis and pubic combing ligament (the extraperitoneal space was not constructed around the pectineus foramina, and there was no clear boundary). The hernia sac was separated after the initial part of the hernia sac was exposed. The hernia sac was dissociated to reach the opening of the inner ring, separated from the spermatic cord and then isolated. The mesh was inserted to shield the opening of the inner ring of the hernia, and the wound was sutured after fixation with glue.

### Observation indicators

All patients were followed up for about half-a-year. The primary outcomes were comparisons of the time of handling hernia, the operation time, intraoperative blood loss, postoperative ambulation time, length of hospital stay, pain at postoperative 24 h and 48 h and the incidence of total complications. The secondary outcomes included peritoneal damage, seroma, inferior epigastric artery injury, sensory nerve abnormalities, chronic pain (lasting over 90 days), incision infection, and hernia recurrence. Pain was evaluated through a visual analog scale (VAS) at 24 h, 48 h and 90 days postoperatively. Range 0–10: a straight line was drawn and marked 1–10, with 0 representing no pain and 10 representing the most severe pain. Patients lasting over 90 days with VAS scores greater than 4 points were considered to have chronic pain.

### Statistical methods

Data were analyzed by SPSS20.0 (SPSS Inc., Chicago, IL, USA). Normal distribution of measurement data, including the age, follow-up time, time of handling hernia, operation time, intraoperative blood loss, postoperative ambulation time, VAS and hospitalization time was checked by Kolmogorov–Smirnov test. Data conforming to normal distribution were presented with mean ± standard deviation and comparisons between groups were conducted by t-test. Otherwise, data were presented with median (interquartile range) and comparisons between groups were conducted by Mann–Whitney U test. Enumeration data, such as location of indirect inguinal hernia, Rutkow classification, ASA classification, size of hernia defect and postoperative complications, are presented as rates and were compared using a chi-square test or Fisher's exact test. The incidence of total complications by follow-up was compared by chi-square test. We used PASS 15 to estimate the sample size. In the preliminary experiment, we found that the total complication rate in procedural group was 4%, and that in the traditional group was 10%. Assuming power of 0.8, alpha was 0.05 and the sample ratio in the procedural group and traditional group was 1:1, the number of samples in each group was calculated to be 45. We assumed the loss of follow-up rate was 10%, and the final estimated number of samples in each group was 50. *P* < 0.05 indicated that the difference was statistically significant.

## Results

**Figure 3:**
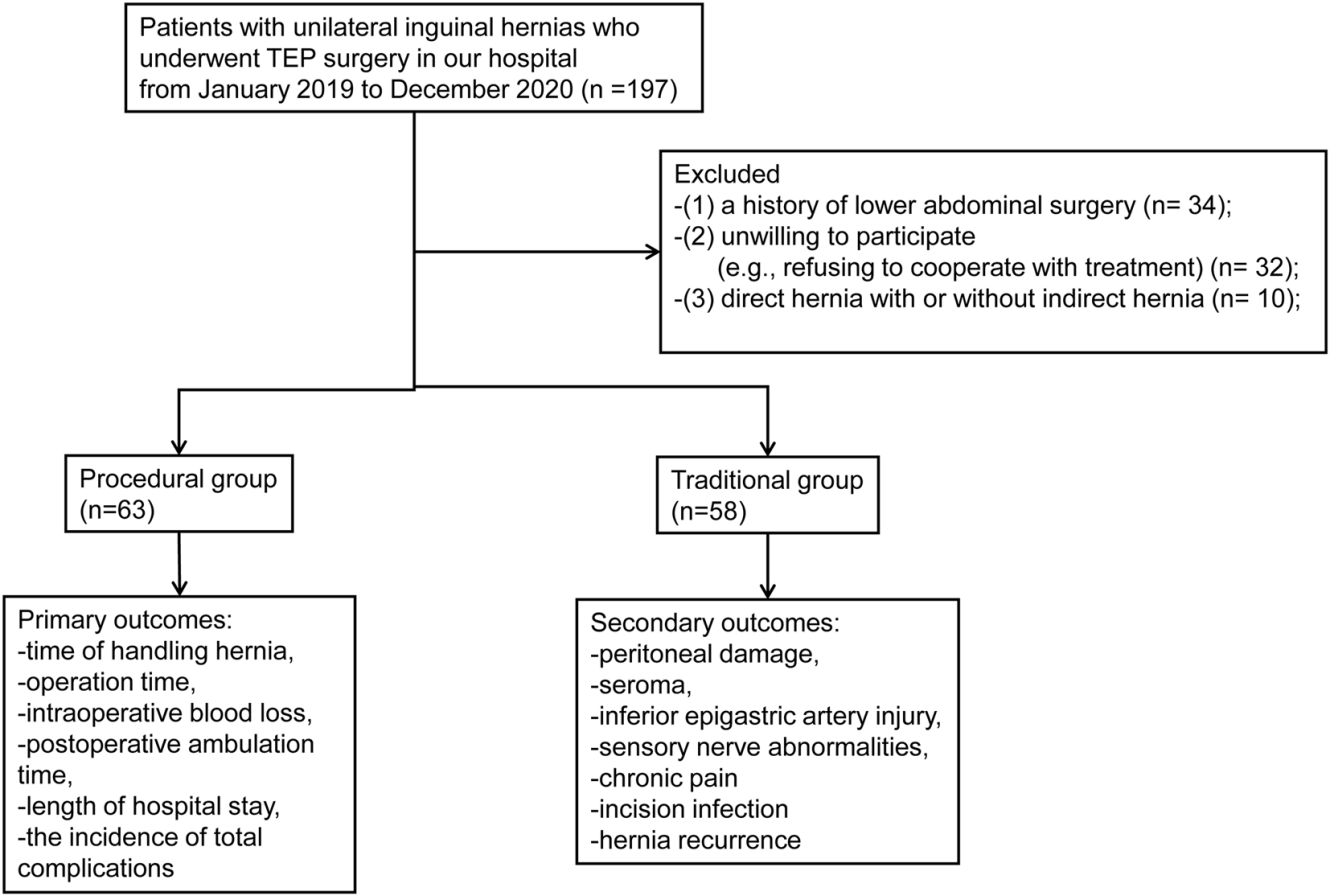
Flow chart of patients’ enrollment.

All patients in the two groups completed the operation, and no patients were converted to other surgery. The flow chart of this study is shown in Fig. 3.

The patients' demographic characteristics (age, location of indirect inguinal hernia, Rutkow Classification, ASA Classification, and size of hernia defect) were carefully extracted and analyzed. The median age of the procedural group was 67 years. The hernia was on the left side in 27 cases and on the right side in 36 cases. According to Rutkow classification, 18 cases were grade I, 36 were grade II, and nine were grade III. There were 48 cases of grade I, 12 of grade II, and three of grade III based on the ASA classification. The median age of the traditional group was 66.5 years. The hernias were on the left side in 19 cases and on the right side in 39. According to Rutkow classification, 10 cases were grade I, 38 were grade II, and 10 were grade III. There were 45 cases of grade I, 11 of grade II, and two of grade III based on the ASA classification. According to the result of ultrasound B, the size of the hernia defects in both groups was undifferentiated. There was no significant difference in baseline demographic characteristics between the two groups (*P* > 0.05; Table 1). The time of follow-up between the procedural group and traditional group is also comparable (*P* > 0.05).

**Table 1.** Baseline demographic characteristics in the traditional and procedural groups.

Characteristics	Traditional group (n = 58)	Procedural group (n = 63)	P-value
Age [median (IQR)] (years)	66.5 (54, 72)	67 (54, 72)	0.950
Location of indirect inguinal hernia (n)			
Left	19	27	0.253
Right	39	36	
Rutkow classification (n)			
Grade I	10	18	> 0.05
Grade II	38	36	
Grade III	10	9	
ASA classification (n)			
Grade I	45	48	0.335
Grade II	11	12	
Grade III	2	3	
Size of hernia defect (cm)			
> 3	18	20	0.933
< 3	40	43	
Follow-up time [median (25% quartile, 75% quartile)] (day)	170.5 (143.25, 192)	182 (182, 182)	0.15

Compared with the traditional group, the time of handling hernia, the operation time, intraoperative blood loss, postoperative ambulation time, postoperative 24 h and 48 h VAS and postoperative hospital stay in the procedural group were significantly decreased (*P* < 0.05; Table 2).

**Table 2:** Comparison of surgical conditions between the traditional group and the procedural group [median (25% quartile, 75% quartile)].

	Traditional group (n = 58)	Procedural group (n = 63)	*P*-value
Time of handling hernia (min)	17.5 (14.75, 21)	11 (8, 13)	< 0.001
Operation time (min)	64 (52.75, 84.25)	55 (50, 65)	0.008
Intraoperative blood loss (ml)	10 (5, 15)	5 (5, 10)	0.002
Postoperative ambulation time (hours)	21.5 (20, 22)	20 (18, 21)	< 0.001
VAS- 24 h	4 (3, 4)	3 (2, 4)	0.011
VAS- 48 h	2 (2, 3)	2 (1, 2)	0.001
Hospital stay (d)	3 (2, 4)	3 (2, 3)	0.001

*VAS* visual analog scale.

The incidence of peritoneal damage was significantly lower than that of the traditional group (*P* = 0.025; Table 3). There were no significant difference between the two groups in other complications, including seroma, inferior epigastric artery injury, sensory nerve abnormalities, and chronic pain(*P* > 0.05; Table 3). Moreover, the incidence of total complications in the procedural group was significantly lower than that of the traditional group (*P* < 0.05).

**Table 3:** Comparison of complications between the traditional group and the procedural group.

	Traditional group (n = 58)	Procedural group (n = 63)	P value
Peritoneal damage [n (%)]	13 (22.4)	5 (7.9)	0.025
Seroma [n (%)]	7 (12.1)	2 (3.2)	0.062
Inferior epigastric artery injury [n (%)]	7 (9.4)	1 (1.6)	0.027
Sensory nerve abnormalities [n (%)]	7 (9.4)	1 (1.6)	0.027
Chronic pain [n (%)]	8 (12.1)	2 (3.2)	0.047
Incision infection [n (%)]	0 (0)	0 (0)	NA
Hernia recurrence [n (%)]	0 (0)	0 (0)	NA
Total complication [n (%)]	25 (43.1)	9 (9.5)	< 0.001

*NA* not applicable.

## Discussion

Inguinal hernia is a relatively common external hernia disease in clinics. With the widespread application of minimally invasive laparoscopic techniques, laparoscopic TEP has become the preferred surgical method for treating inguinal hernias. Compared with traditional laparotomy, TEP is characterized by smaller surgical incisions, less pain, faster postoperative recovery, and a lower complication rate, which conforms to the high medical needs of modern patients^7^.

With the continuous improvement of medical technology and physician skills, the proposal of TEP surgery based on the spatial anatomy of the myopectineal orifice has attracted increasing attention and has become a hot spot in the field of endoscopic inguinal hernia treatment^8,9^. Traditional TEP follows the guiding concept of open inguinal hernia surgery. Only the operative approach and operative field of view are changed, and no adjustment is made to the hierarchical structure of the myopectineal orifice.

Due to the unclear spatial concept of extraperitoneal space establishment and unorganized surgical procedures, it is difficult to completely discover the myopectineal orifice, which significantly increases the probability of intraoperative vascular injury and the difficulty of surgical operation, thus hindering the promotion of TEP. The introduction of the surgical operation concept of "programmed spatial anatomy of the myopectineal orifice" can systematically and hierarchically divide the extraperitoneal space into four spatial areas, and clarify the operational focus and difficulties in each spatial area, making the surgical operation clear and organized, saving intraoperative operation time, and reducing the incidence of intraoperative and postoperative complications. Meanwhile, this method simplifies and visualizes the anatomical knowledge of the myopectineal orifice, which is easy to remember and understand and significantly shortens the learning curve for young surgeons.

**Figure 4:**
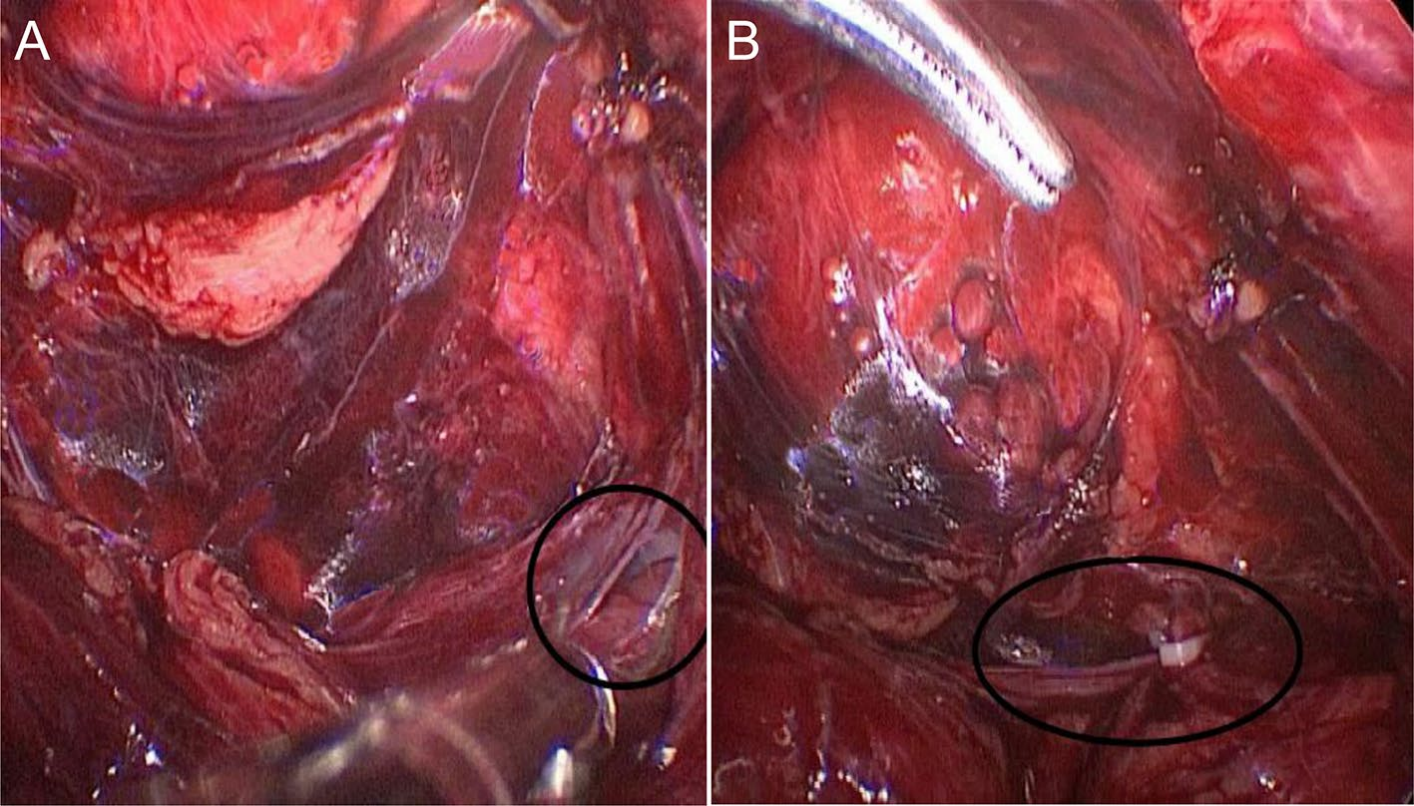
Treatment of peritoneal damage. Breaks larger than 2 cm can be closed with absorbable clips before surgery.

Establishing sufficient space is an important factor for the success of TEP surgery. In the process of "programmed spatial anatomy of the myopectineal orifice," by using the myopectineal orifice as the center, a space can be fully established between the deep layer of the transverse abdominal fascia and the peritoneum. Thus, the plastic three-dimensional (3D) mesh can be spread flat enough to effectively block the myopectineal orifice area and reduce the occurrence of curling and folding during the spreading process. The peritoneum may be damaged when the patient has a history of abdominal wall infection, which leads to dense adhesion of the space or difficulty in the dissection of the hernia sac. Generally, the operation can be completed if the rupture is smaller than 2 cm. If the rupture was larger than 2 cm in our study, the incision was first closed with an absorbable clip and then operated on (Fig. 4). Laparoscopic transabdominal preperitoneal inguinal hernia repair (TAAP) may be used in cases where it is difficult to close the incision, which may affect TEP surgery.

The "programmed spatial anatomy of the myopectineal orifice" can observe important anatomical landmark structures in each area and effectively separate them, significantly improving TEP surgery's success rate. The establishment of the "first space" establishes a flat surface for surgery. If this space is not handled correctly, it is likely to cause a rupture into the abdominal cavity and force the transformation into TAAP.

It is recommended to make the observation incision at the lower umbilicus of the affected side, which is conducive to exposing the posterior sheath structure of the rectus abdominis and constructing a correct flat surface. When establishing the "first space," the laparoscopic lens is pushed forward along the posterior sheath of the rectus abdominis to initially and quickly expose the preperitoneal space^9,10^. The key points during the establishment of the "second space" are to fully separate the Retzius space and protect the important anatomical structures. The lens pushing method can initially open this space, and structures such as the pubic symphysis and Cooper's ligament can sometimes be displayed. If the tissue is dense, an electric hook can be used to carefully separate the Retzius space and search the direct hernia triangle, the crown of death, and the inferior epigastric artery in the upper half of the medial myopectineal orifice.

During the establishment of this space, Cooper's ligament and the inferior epigastric artery are regarded as the relevant landmarks for separating the peritoneum. The superficial layer of the transverse abdominal fascia should not be damaged to avoid small venous hemorrhage or corona mortis and inferior epigastric artery damage due to excessive separation, thus resulting in massive hemorrhaging. Generally, it is advisable not to fold the 3D mesh when placing it under the pubic tubercle. After the "second space" is fully established, the lateral border of the myopectineal orifice area is separated outward along the direction of the inferior epigastric artery, and then the "third space" is entered.

Posterior to the inferior epigastric artery is the Bogros space, which is loose and easily separated. The main anatomical structures of the Bogros space are the internal ring of the indirect inguinal hernia, the hernia sac, and the pain triangle. During the separation process, the space on both sides of the hernia sac should first be fully freed to isolate the hernia sac, which is beneficial to the subsequent dissection and operation of the "fourth space." When separating the Bogros space, attention should be paid to protecting the pain triangle to avoid damage to the lateral cutaneous and genitofemoral nerves within the triangle, resulting in postoperative pain and discomfort in patients in the later stage. The hernia sac is carefully stripped from the vas deferens and spermatic vessels and is dissociated downwards, which is the process of establishing the "fourth space."

Blunt dissection is performed gently to achieve deperitonealization of the spermatic cord using the peritoneal line at the lower border of the myopectineal orifice as the baseline. Care should be taken not to damage the danger triangle during separation. Meanwhile, the "programmed spatial anatomy of the myopectineal orifice" completes the establishment of the preperitoneal space. The 3D mesh is then placed, and the preperitoneal space can be effectively covered without any fixation after a little adjustment. The mesh will fit the abdominal wall without displacement after the operation. There is no need to fix the mesh with a nail gun or glue at the markers of each spatial anatomical feature.

Our results showed that compared with the traditional group, the time of handling hernia, the operation time, intraoperative blood loss, postoperative ambulation time, postoperative 24 h and 48 h VAS and postoperative hospital stay in the procedural group were significantly decreased. These results indicated that the programmed spatial anatomy of the myopectineal orifice for TEP surgery could effectively improve the efficacy of surgical treatment and accelerate the postoperative recovery of patients. The key to the success of programmed spatial anatomy of the myopectineal orifice is to accurately determine the spatial location of the pectineal orifice during surgery, effectively divide the spatial area and accurately locate the surgical operation. In the comparison of postoperative complications, procedural group could effectively reduce the incidence of postoperative complications of TEP, including either short-term complications or long-term complications. Moreover, the incidence of total complications, which reflects the overall postoperative recovery of patients, in the procedural group was significantly lower than that of the traditional group. This result suggested that TEP surgery based on programmed spatial anatomy of the myopectineal orifice has higher safety. This is because the intraoperative damage to the relevant nerves and blood vessels is reduced due to the clear operation procedure and evaluation index, thus the incidence of postoperative complications is reduced.

The "programmed spatial anatomy of the myopectineal orifice" has practical clinical significance for young physicians. Judging from the effect of the technique’s promotion in our hospital, this technology allows young physicians to accurately determine the specific location of the myopectineal orifice and clarify the meaning of each anatomical landmark and possible intraoperative conditions through the effective division of each space. Thus, intraoperative bleeding and accidental nerve injury can be effectively avoided. The accuracy of surgery can be significantly improved after mastering this technique, demonstrating the advantages of the minimally invasive concept.

However, this study also has some limitations. First, it is a retrospective observational study, and further prospective randomized controlled trials are needed to further strengthen our conclusion and provide better evidence for clinical practice. Second, it is only a single-center study involving a small number of cases. Large -scale multicenter research may be needed for confirmation.

In conclusion, applying the "programmed spatial anatomy of the myopectineal orifice" surgical technique is safe and reliable. It can significantly improve the clinical effect of TEP hernia repair and intra- and postoperative clinical indicators and reduce the incidence of intra- and postoperative complications. In addition, it can improve the practical clinical ability of young doctors, shorten the TEP learning cycle, and facilitate grass-roots promotion.

## Data Availability

The datasets used and/or analysed during the current study available from the corresponding author on reasonable request.
