# Author Correction: Therapeutic efficacy of programmed spatial anatomy of the myopectineal orifice in total extraperitoneal hernioplasty: a retrospective study

**DOI:** 10.1038/s41598-023-31324-1

**Published:** 2023-03-21

**Authors:** Lin Zhuang, Yuanjiu Li, Wei He, Xiaodong Zhou, Yan Chen, Xiaozhong Wang, Bo Wang, Xuezhong Xu, Kejia Wu, Qiutao Zhang, Dong Xi, Yunjie Lu

**Affiliations:** 1 Department of General Surgery, Wujin Affiliated Hospital of Jiangsu University and The Wujin Clinical College of Xuzhou Medical University, Changzhou, Jiangsu China; 2 The First People’s Hospital of Changzhou, The Third Affiliated Hospital of Soochow University, Changzhou, Jiangsu China; 3 Department of General Surgery, Wujin Fourth People’’s Hospital, Changzhou, Jiangzhou China; 4 Africa Hepatopancreatobiliary Cancer Consortium (AHPBCC), Mayo Clinic, Jacksonville, FL USA

Correction to:* Scientific Reports* https://doi.org/10.1038/s41598-023-29671-0, published online 15 February 2023

The original version of this Article contained errors in the Affiliations.

Qiutao Zhang was incorrectly affiliated with ‘The First People’s Hospital of Changzhou, The Third Affiliated Hospital of Soochow University, Changzhou, Jiangsu, China’ and ‘Africa Hepatopancreatobiliary Cancer Consortium (AHPBCC), Mayo Clinic, Jacksonville, FL, US’.

The correct affiliation for Qiutao Zhang is listed below.

Department of General Surgery, Wujin Affiliated Hospital of Jiangsu University and The Wujin Clinical College of Xuzhou Medical University, Changzhou, Jiangsu, China.

Additionally, Yunjie Lu was incorrectly affiliated with ‘Department of General Surgery, Wujin Affiliated Hospital of Jiangsu University and The Wujin Clinical College of Xuzhou Medical University, Changzhou, Jiangsu, China.’

The correct affiliations for Yunjie Lu are listed below.

The First People’s Hospital of Changzhou, The Third Affiliated Hospital of Soochow University, Changzhou, Jiangsu, China.

Africa Hepatopancreatobiliary Cancer Consortium (AHPBCC), Mayo Clinic, Jacksonville, FL, US.

The original Article has been corrected.

